# Effect of depression, anxiety, and distress screeners on the need, intention, and utilization of psychosocial support services among cancer patients

**DOI:** 10.1007/s00520-023-07580-2

**Published:** 2023-01-16

**Authors:** Franziska Springer, Leon Sautier, Georgia Schilling, Uwe Koch-Gromus, Carsten Bokemeyer, Michael Friedrich, Anja Mehnert-Theuerkauf, Peter Esser

**Affiliations:** 1 Department of Medical Psychology and Medical Sociology, University Medical Center Leipzig, Philipp-Rosenthal-Str. 55, 04103 Leipzig, Germany; 2 Department of Medical Psychology, University Medical Center Hamburg Eppendorf, Hamburg, Germany; 3 Department of Clinical Oncology, Asklepios Tumorzentrum Hamburg, Hamburg, Germany; 4 Department of Internal Medicine II, University Medical Center Hamburg-Eppendorf, Hamburg, Germany

**Keywords:** Cancer, Psycho-oncology, Psychosocial needs, Health care utilization, Screening, Distress

## Abstract

**Purpose:**

In clinical cancer care, distress screening is recommended to identify highly burdened patients in objective need for psychosocial support to improve psychological distress and quality of life and to enhance patient empowerment. It is however unclear whether distress screeners are suitable for psychosocial care planning and thus whether they can predict the willingness that is *need*, *intention*, and *utilization*, to seek psychosocial support.

**Methods:**

In a secondary analysis of a cluster intervention study, we assessed cancer patients with three distress screeners (DT, PHQ-9, GAD-7) at baseline. The willingness to seek psychosocial support services was assessed binary for psychosocial services at 3 and 6 months. Logistic regression models were applied to examine the predictive effect of the screeners on need, intention, and utilization. We corrected all models for multiple testing.

**Results:**

The 660 patients included in the study were on average 60 years, 54% were male. At the 3- and 6-month follow-up, 353 and 259 patients participated, respectively. The screeners were best in predicting the need for support (OR reaching up to 1.15, 1.20, and 1.22 for the PHQ-9, GAD-7, and DT respectively). The intention was predicted by the PHQ-9 and GAD-7, whereas utilization of psychosocial support services was not predicted by the screeners.

**Conclusion:**

The three distress screeners might be useful in psychosocial care planning, as they are able to predict the need and to some degree the intention to seek psychosocial support. Future research needs to examine potential barriers and supporting factors that may explain utilization of psychosocial support.

**Trial registration:**

The study was retrospectively registered (2/2021) at ClinicalTrials.gov (number: NCT04749056).

**Supplementary Information:**

The online version contains supplementary material available at 10.1007/s00520-023-07580-2.

## Introduction

Cancer patients face multiple challenges during their disease and about 50% suffer from significant levels of distress [1, 2]. Besides distress, many patients experience symptoms of anxiety, depression [3], or fear of cancer progression [4]. Regular distress screening is recommended in cancer care [5] to identify patients who are highly distressed and to offer psychosocial support by health care professionals. Established screening instruments such as the *Distress Thermometer* (DT) [6], the *Patient Health Questionnaire* (PHQ-9) [7], or the *General Anxiety Disorder Scale* (GAD-7) [8] are developed and well validated for the screening of the severity of psychological symptoms. Guidelines, e.g. by the American Society of Clinical Oncology (ASCO) [9], use cut-offs on these established distress screeners to recommend further actions. Screeners are often used in clinical care to identify distressed patients and to plan psychosocial support offers, even though they were not originally developed for this. The extent to which these screeners are suitable for clinical care planning and can predict the need, the intention, and the utilization of psychosocial support services, however, is poorly discussed in the literature. This would highly be important to facilitate appropriate care planning.

Cancer patients with higher level of distress and anxiety have been found to report increased subjective care needs [10–14]. However, the informative value of previous research is limited. A study of 302 cancer patients [11] used only single items to assess needs and distress, which reflects only a rough estimate and thus limits the validity of the findings and hamper a comparison of different screenings. The pilot study for the current project with 335 cancer patients [12] indeed investigated the association between different screeners for anxiety (GAD-7 and FOP-Q-SF [15]) and types of psychosocial needs, but was cross-sectional in design, which does not permit prediction of future needs.

Inconsistent results are reported regarding the association of distress screeners and utilization of psychosocial care [12, 16–19]. A methodologically similar study to ours found an association between the DT, PHQ-9, and GAD-7 screeners and the use of psychological support [19], but was again designed cross-sectional. However, if distress screeners are able to predict the complex decision of seeking support is questionable. This aspect needs further clarification using longitudinal data.

To interpret the value of the screeners for care planning, a distinction between different dimensions of willingness to seek psychosocial support seems necessary, that is the *need*, the *intention*, and the actual *utilization* of psychosocial support services. Only few studies investigated more than one outcome and pointed to a differentiated pattern: one study found distress to be related with care needs, but not to higher utilization of psychosocial support [12], another found distress to be related to intention, but not utilization [20].

In summary, previous relevant research is sparse, mostly cross-sectional and does not allow for a systematic comparison of screeners and dimensions of willingness. The objective of the current study was to investigate the predictive value of three established distress screeners on the dimensions of willingness to seek psychosocial support in a secondary analysis of a longitudinal study. Findings may help to establish hypotheses whether and which screeners may be best suited for appropriate psycho-oncological care planning.

## Methods

### Study design

The data for this secondary analysis was obtained from a controlled cluster intervention study evaluating a psycho-oncological online-screening program among cancer patients [21]. The intervention group received a tablet-based adaptive screening application, which provides immediate feedback and recommendations for psychosocial support at the UCCH. Physicians were further recommended to talk to patients if they were highly distressed based on the screening results. The control group had the same access to psychosocial support services at UCCH as the intervention group but did not receive any feedback on their assessment. The intervention was not associated with any of the measures (screeners) used in the current study [21].

### Participants

Patients were consecutively recruited by trained research assistants in eleven urban inpatient and outpatient cancer care facilities at the University Cancer Center Hamburg (UCCH). Eligibility criteria were checked via review of the medical charts by research assistants. Patients were eligible if they were (i) diagnosed with cancer according to ICD-10, (ii) ≥ 18 years, (iii) fluent in German language, and (iv) currently accessible for the research assistants (i.e., they were excluded if they were in isolation due to medical reasons). Inclusion and exclusion criteria were the same in the main analysis. Written informed consent was provided to the research assistants by all participants prior to the study. The study protocol was approved by the ethics committee of the medical chamber of Hamburg (PV4371) and was retrospectively registered (2/2021) at ClinicalTrials.gov (number: NCT04749056).

### Data collection

At baseline (*t*0), all participants completed a questionnaire in the respective care facility either online (intervention group, combined with the online intervention program) or paper-pencil (control group). Follow-up questionnaires after 3 months (*t*1) and 6 months (*t*2) were then sent to all participants paper-pencil by mail to fill in at home. Participants were reminded after 2 weeks if the questionnaire was not returned.

### Screeners

#### Depression screener

We used the PHQ-9 [7], a 9-item self-report questionnaire based on the DSM-IV criteria. Values range from 0 to 27. A higher value indicates stronger depressive symptoms. It shows good reliability and validity [7] and is recommended as a screening instrument for depression in cancer patients [22]. Based on current guidelines [9], a cut-off ≥8 is recommended to have further diagnostic assessment to identify the nature and extend of depressive symptoms and to consider a referral.

#### Anxiety screener

The GAD-7 [8] is a 7-item self-report questionnaire to assess anxiety symptoms. Values range from 0 to 21. A higher score indicates stronger anxiety symptoms. It shows good reliability and validity [8] and is recommended in clinical practice to identify patients with general anxiety disorder [23]. A cut-off ≥10 is recommended to have further diagnostic assessment and to consider a referral [9].

#### General distress screener

The DT is a well-established one-item scale ranging from 0 (no distress) to 10 (extreme distress), which is recommended to be used routinely in clinical care [6, 24]. The cut-off ≥5 indicates a clinically relevant level of distress [25]. The 1-item screening tool shows good reliability and validity.

### Dimensions of willingness to seek psychosocial support

Three dimensions of willingness to seek psychosocial support were assessed in the study: general *need*, *intention* to use, and actual *utilization* of psychosocial support. The need was assessed with ten items assessing each of the ten psychosocial support services available at the UCCH rated on a binary item (yes/no). For this study which focused on emotional distress, we selected only those support services that directly addressed psychosocial and psycho-oncological issues, i.e., (i) social service (psychosocial and social-law consulting), (ii) psycho-oncological service, and (iii) survivorship program. Analogously to the need, we assessed the respective intention and utilization of support services (yes/no).

### Sociodemographic and medical data

Sociodemographic data were provided by self-report. All medical data were extracted from the medical chart information.

### Statistical analyses

Sociodemographic and medical data were analyzed descriptively. Drop-out analyses were conducted at baseline via Chi-square- and *t*-test. Missing values of the screeners were replaced with mean imputation, whereas missing values at the dimensions of willingness were not imputed and remained missing. To investigate the predictive effects of the three distress screeners at baseline on the willingness to seek psychosocial support at *t*1 and *t*2, we applied several unconditional logistic regression models. In detail, we separately analyzed the predictive effect of each screener (PHQ-9, GAD-7, DT) on the dimensions of willingness (need, intention, utilization), for each type of service (social service, psycho-oncological service, survivorship program) at each time point (*t*1, *t*2). Thus, a total of 54 (3×3×3×2) models were performed here. All 54 analyses were recalculated by using the cut-offs of the screeners which have been suggested in previous guidelines [9, 25] (PHQ-9≥8, GAD-7≥10, DT≥5), to investigate their usefulness in care planning. The regression models were calculated by controlling for the intervention in order to compensate for the intervention effect as well as age and gender. To assess the robustness of the findings of the main analysis, several sensitivity analyses were performed: for the control group only, to rule out the influence of the intervention on the overall conclusion, and for patients with curative and palliative treatment intention separately, to examine the predictive effect for the central medical variable in drop-outs.

All regression models were adjusted for the alpha-level by using Bonferroni-Holm correction [26]. Adjustment was made according to the central outcome dimension, i.e. the three dimensions of willingness. According to the regression models of the screeners, *p*-values were adjusted for 18 models for each dimension of willingness (3 screeners × 3 psychosocial support services × 2 time points). No adjustment was made for the sensitivity analyses due to the reduced sample size. A comparison between the main analysis and the sensitivity analyses was consequently made with unadjusted alpha-values to compare the pattern of results.

Effect sizes for all logistic models were reported as odds-ratio (OR) that may range from 0 to infinite [27]. Due to differences in the scaling of the three screeners, a comparison of the ORs between screeners is not possible.

All analyses were conducted using R (version 4.1.0).

## Results

### Participant flow

Data collection took place from December 2013 to July 2015. A total of 1784 participants were checked for eligibility, of which 1320 were eligible. Most common reasons for ineligibility were severe physical/mental/cognitive impairment (*n*=323) and insufficient language skills (*n*=91). In total, 660 out of 1320 (50%) eligible patients were included in the study. Most common reasons for non-participation were no interest (*n*=237) and physical/mental burden (*n*=46). Furthermore, some patients were excluded due to technical problems or incapability to use the EPAS program (*n*=33) and too few questionnaire data available (*n*=198). For more details on participant flow, see previous publication [21].

In the follow-up, 353 patients participated at *t*1 (3-month follow-up, drop-out rate: 47%) and 259 participated up to *t*2 (6-month follow-up, drop-out: rate 61%). Patient loss on follow-up was mostly due to not sending back the questionnaires and deceased patients.

Compared to study completers, dropouts were more likely to receive palliative treatment (*p*<.001), have a relapse (*p*=.014), a longer time since diagnosis (*p*=.042), and no partner (*p*=.037) (data not shown). Furthermore, dropouts showed a higher level of depressive symptomatology (PHQ-9) at baseline (*p*=.003), whereas no differences were found for GAD-7 and DT.

### Baseline sample characteristics

On average, patients were 60 years old and 54% were male. About half of the patients received their diagnosis within the last 3 months and the most frequent cancer types were hematological cancer (32%) and colorectal cancer (12%). Fifty-four percent of the patients had a palliative treatment intention (Table 1).

**Table 1:** Baseline sample characteristics

		*n* **(%)**
Age in years	Mean (SD)	60 (13.6)
Gender	Female	300 (46)
Partnership	Yes	363 (59)
Education	< 10 years at school	176 (28)
	≥ 10 years at school	484 (72)
Cancer type	Hematologic (C81-86, C90-93, C95, D46)	211 (32)
	Colorectal (C17-21)	77 (12)
	Lung (C34)	68 (10)
	Breast (C50)	68 (10)
	Head and neck (C00-02, 04, 07-11, 13/14, 30, 32, 76)	35 (5)
	Stomach/Eosophagus (C15/16)	30 (5)
	Prostate (C61)	25 (4)
	Pancreas (C25)	23 (4)
	Gall/liver (C22, 24)	15 (2)
	Female genital organs (C53/54, 56/57)	17 (3)
	Urogenital (C64, 67/68)	13 (2)
	Testis (C62)	13 (2)
	Others	65 (10)
Treatment setting	Outpatient	375 (57)
	Inpatient	285 (43)
Months since current diagnosis (Mean, SD)		12 (27)
	≤ 3 months	337 (52)
	> 3 months	312 (48)
Treatment intention	Curative	280 (46)
	Palliative	328 (54)
TNM-T^a^	0: no tumor/CUP	7 (2)
	1: < 2 cm	43 (11)
	2: 2–5 cm	73 (19)
	3: > 5 cm	119 (31)
	4: ext. to skin/chest wall	61 (16)
	X: not assessable	82 (21)
TNM-N^b^	0: no lymph node metastases	94 (24)
	1–3: lymph node metastases	189 (49)
	X: not assessable	102 (27)
TNM-M^c^	0: no distant metastases	147 (38)
	1: distant metastases	236 (61)
	X: no information	2 (1)
UICC^d^	I	25 (6)
	II	41 (9)
	III	76 (17)
	IV	243 (56)
	Not evaluable	52 (12)
Type of treatment	Surgery	255 (39)
	Radiation	176 (27)
	Chemotherapy	591 (90)
	Pharmacological	49 (7)
	Stem cell or bone marrow transplantation	24 (4)
	Other	143 (22)
PHQ-9^g^	Mean (SD)	7.6 (4.9)
	Sum score ≥ 8	276 (44)
GAD-7^h^	Mean (SD)	4.6 (4.1)
	Sum score ≥ 10	76 (12)
DT^i^	Mean (SD)	5.0 (2.5)
	≥ 5	364 (59)

Since this is a secondary analysis, this table has already been published in a very similar form [21]; all values based on t0 sample (*N=*660) if not otherwise stated; SD, standard deviation; ^a^size and extent of the tumor, excluding hematological caner; ^b^absence or presence and extent of regional lymph node metastasis, excluding hematological cancer; ^c^the absence or presence of distant metastasis, excluding hematological cancer; ^d^excluding hematological cancer; ^g^PHQ-9 at baseline, sum score according to existing ASCO guidelines: none to mild symptomatology: <8, moderate to severe symptomatology: ≥8; ^h^GAD-7 at baseline, sum score according to existing ASCO guidelines: none to mild symptomatology: <10, moderate to severe symptomatology: ≥10; ^i^DT at baseline, clinically relevant level of distress: ≥5

### Predictive effects across dimensions of willingness

All three screeners most often predicted the need for the psychosocial support, with respective OR reaching up to 1.15, 1.20, and 1.22 for the PHQ-9, GAD-7, and DT (Table 2). Effects on the intention for usage were predicted in fewer cases and only by the PHQ-9 and GAD-7, with OR for both screeners reaching up to 1.15. The actual utilization of psychosocial support was not predicted by any of the screeners. The service that was most often predicted by the screeners was psycho-oncological service, followed by social service.

**Table 2:** Predictive effect of the three screeners as sum score values on service use by dimensions of willingness and types of services at *t*1 and *t*2

		*t*1 (3-month follow-up)				*t*2 (6-month follow-up)			
		*N*	*Β (SE)*	*p*^*a*^	*OR*	*N*	*Β (SE)*	*p*^*a*^	*OR*
Need									
PHQ-9	*Social service*	313	0.10 (0.03)	**<.001**	1.11	212	0.14 (0.03)	**<.001**	1.15
	*Psycho-onco*	313	0.09 (0.03)	**<.001**	1.10	217	0.13 (0.03)	**<.001**	1.14
	*Survivorship*	313	0.09 (0.03)	**.003**	1.09	208	0.04 (0.04)	.360	1.04
GAD-7	*Social service*	314	0.11 (0.03)	**<.001**	1.12	214	0.18 (0.04)	**<.001**	1.19
	*Psycho-onco*	314	0.13 (0.03)	**<.001**	1.13	219	0.18 (0.04)	**<.001**	1.20
	*Survivorship*	314	0.08 (0.04)	.028	1.08	209	0.11 (0.05)	.017	1.12
DT	*Social service*	308	0.16 (0.06)	**.006**	1.17	206	0.18 (0.07)	.017	1.19
	*Psycho-onco*	308	0.19 (0.06)	**.002**	1.21	211	0.20 (0.07)	**.006**	1.22
	*Survivorship*	308	0.17 (0.07)	.012	1.19	202	0.05 (0.09)	.559	1.05
Intention									
PHQ-9	*Social service*	291	0.07 (0.03)	.005	1.08	199	0.10 (0.04)	**.003**	1.11
	*Psycho-onco*	291	0.09 (0.03)	**<.001**	1.10	202	0.14 (0.04)	**<.001**	1.15
	*Survivorship*	289	0.08 (0.03)	.008	1.08	200	0.08 (0.04)	.024	1.08
GAD-7	*Social service*	292	0.09 (0.03)	.004	1.09	201	0.12 (0.04)	**.003**	1.13
	*Psycho-onco*	292	0.09 (0.03)	.004	1.10	204	0.14 (0.04)	**<.001**	1.15
	*Survivorship*	290	0.07 (0.03)	.032	1.07	201	0.10 (0.04)	.018	1.10
DT	*Social service*	286	0.02 (0.01)	.143	1.02	193	0.05 (0.08)	.501	1.05
	*Psycho-onco*	286	0.07 (0.06)	.240	1.08	196	0.19 (0.08)	.014	1.20
	*Survivorship*	284	0.13 (0.07)	.050	1.14	194	0.09 (0.08)	.276	1.09
Utilization									
PHQ-9	*Social service*	322	0.05 (0.03)	.084	1.05	237	0.02 (0.03)	.537	1.02
	*Psycho-onco*	324	0.07 (0.03)	.006	1.08	236	0.06 (0.03)	.050	1.07
	*Survivorship*	314	−0.12 (0.10)	.216	0.88	235	0.01 (0.12)	.948	1.01
GAD-7	*Social service*	323	0.02 (0.03)	.503	1.02	239	0.06 (0.04)	.116	1.06
	*Psycho-onco*	325	0.08 (0.03)	.010	1.09	238	0.09 (0.04)	.015	1.10
	*Survivorship*	315	−0.12 (0.12)	.342	0.89	237	0.05 (0.13)	.690	1.05
DT	*Social service*	317	0.12 (0.06)	.042	1.12	231	0.13 (0.07)	.056	1.14
	*Psycho-onco*	319	0.17 (0.06)	.005	1.19	230	0.24 (0.08)	.003	1.27
	*Survivorship*	309	−0.12 (0.17)	.473	0.88	229	−0.10 (0.27)	.714	0.91

All regressions are controlled for intervention, age, and gender; *B*, unstandardized regression coefficient; *SE*, standard error; *p*, unadjusted *p*-value of the regression model; *OR*, odds-ratio; ^a^Bonferroni-Holm adjusted alpha-level *α*_adjusted_=0.05/(18−*k*+1) with *k*=rank number of *p*-value, significant *p*-values after adjustment are marked in bold

### Predictive effects across screeners

Considering all types of support services, dimensions of willingness, and time points, the PHQ-9 and the GAD-7 predicted the willingness to use psychosocial support more often than the DT (Table 2). OR reached up to 1.15, 1.20, and 1.22 for the PHQ-9, GAD-7, and DT, respectively.

### Predictive effects using cut-offs to define clinical relevance

Analyzing the cut-offs of the screeners to indicate their clinical relevance yielded a similar pattern of results to those obtained from the analyses including the continuous data of the screeners (Table 3). That is, the need for psychosocial support was most often predicted, followed by the intention to use these support services. Again, the screeners did not predict the actual utilization of psychosocial support. Moreover, the PHQ-9 and GAD-7 predicted the willingness to use psychosocial support more often than the DT, as in this case the DT did not predict any dimension of willingness. ORs for the need and intention to use psychosocial support ranged from 2.26 to 3.44 and from 3.34 to 5.55 for the PHQ-9 and the GAD-7, respectively.

**Table 3:** Cut-off predictive effect of the three screeners on service use by dimensions of willingness and types of services at *t*1 and *t*2

		*t*1 (3-month follow-up)				*t*2 (6-month follow-up)			
		*N*	*Β (SE)*	*p*^*b*^	*OR*	*N*	*Β (SE)*	*p*^*b*^	*OR*
Need									
PHQ-9^a^	*Social Service*	313	0.82 (0.26)	**.002**	2.26	212	1.15 (0.33)	**<.001**	3.17
	*Psycho-onco*	313	0.74 (0.27)	.006	2.10	217	1.24 (0.33)	**<.001**	3.44
	*Survivorship*	313	0.63 (0.31)	.043	1.87	208	0.18 (0.41)	.662	1.19
GAD-7^a^	*Social Service*	314	0.76 (0.37)	.038	2.14	214	1.43 (0.45)	**.002**	4.17
	*Psycho-onco*	314	1.35 (0.37)	**<.001**	3.87	219	1.71 (0.46)	**<.001**	5.55
	*Survivorship*	314	0.55 (0.41)	.184	1.73	209	0.71 (0.53)	.180	2.03
DT^a^	*Social Service*	308	0.56 (0.28)	.043	1.76	206	0.27 (0.33)	.418	1.30
	*Psycho-onco*	308	0.60 (0.29)	.039	1.82	211	0.45 (0.34)	.177	1.57
	*Survivorship*	308	0.64 (0.34)	.057	1.90	202	0.18 (0.41)	.653	1.20
Intention									
PHQ-9^a^	*Social Service*	291	0.53 (0.27)	.047	1.70	199	1.11 (0.34)	**.001**	3.02
	*Psycho-onco*	291	0.78 (0.29)	.006	2.18	202	1.16 (0.33)	**<.001**	3.20
	*Survivorship*	289	0.75 (0.30)	.011	2.12	200	0.42 (0.36)	.242	1.52
GAD-7^a^	*Social Service*	292	0.98 (0.37)	.007	2.67	201	0.49 (0.47)	.295	1.63
	*Psycho-onco*	292	1.21 (0.37)	**.001**	3.34	204	1.14 (0.45)	.011	3.11
	*Survivorship*	290	0.43 (0.40)	.280	1.54	201	0.40 (0.50)	.419	1.49
DT^a^	*Social Service*	286	0.07 (0.27)	.805	1.07	193	-0.01 (0.33)	.980	0.99
	*Psycho-onco*	286	0.23 (0.30)	.437	1.26	196	0.59 (0.34)	.086	1.80
	*Survivorship*	284	0.55 (0.31)	.078	1.74	194	0.68 (0.37)	.064	1.98
Utilization									
PHQ-9^a^	*Social Service*	322	0.44 (0.26)	.089	1.55	237	0.24 (0.31)	.439	1.27
	*Psycho-onco*	324	0.54 (0.27)	.047	1.71	236	0.62 (0.34)	.065	1.86
	*Survivorship*	314	-0.60 (0.84)	.480	0.55	235	0.08 (1.27)	.947	1.09
GAD-7^a^	*Social Service*	323	0.17 (0.38)	.656	1.19	239	0.39 (0.44)	.384	1.47
	*Psycho-onco*	325	0.76 (0.37)	.043	2.13	238	0.57 (0.46)	.212	1.78
	*Survivorship*	315	-6.82 (28.54)	.811	0.00	237	1.36 (1.26)	.280	3.89
DT^a^	*Social Service*	317	0.52 (0.27)	.055	1.69	231	0.40 (0.31)	.203	1.49
	*Psycho-onco*	319	0.75 (0.30)	.012	2.11	230	0.93 (0.36)	.011	2.54
	*Survivorship*	309	-0.57 (0.79)	.473	0.57	229	-0.82 (1.26)	.513	0.44

All regressions are controlled for intervention, age, and gender; *B*, unstandardized regression coefficient; *SE*, standard error; *p*, unadjusted *p*-value of the regression model; *OR*, odds-ratio; ^a^based on cut-offs according to existing guidelines (PHQ-9 cut-off ≥8; GAD-7 cut-off ≥10; DT cut-off ≥5); ^b^Bonferroni-Holm adjusted alpha-level *α*_adjusted_=0.05/(18−*k*+1) with *k*=rank number of *p*-value, significant *p*-values after adjustment are marked in bold

### Predictive effects across measurement points

In general, the screeners predicted the willingness to seek psychosocial support more often at *t*2 than at *t*1 (Table 2, Table 3). Specifically, more effects reached significance at *t*2 and effects that were significant at *t*1 also became significant at *t*2 (except for three).

### Sensitivity analysis

The sensitivity analysis of the control group (Table S1) and of patients with curative (Table S2) and palliative (Table S3) treatment intention revealed a similar pattern of results as the main analysis.

## Discussion

### Main findings

This secondary analysis among a sample of cancer patients investigated the predictive effect of three established distress screeners on different dimensions of willingness to seek psychosocial support. The screeners were best in predicting the need for psychosocial support, whereas none of the screeners predicted the actual utilization. The service most often predicted by the screeners was psycho-oncological service. The PHQ-9 and GAD-7 predicted the willingness to seek psychosocial support more often than the DT did.

### Integration into previous research

Our data revealed an association between the need for psychosocial support and the PHQ-9 and GAD-7. Consistent with our findings, a cross-sectional study with 4091 patients with heterogeneous cancer diagnoses reported an increase in psychosocial needs in patients with elevated levels on the PHQ-9 above the cut-off [28]. The association between the GAD-7 as sum score and psychosocial needs was also reported in the pilot study to this project with a comparable sample in regard to tumor types, time since diagnosis, and disease status [12]. The PHQ-9 sum score and the GAD-7 cut-off were not examined in previous studies in relation to need to compare our results. Our data implies that both screeners are applicable in this context as a sum score and as cut-off screener. We further found an association between the DT score and psychosocial needs, but not when analyzing the cut-off (≥5). This is in contrast to other studies, which reported patients with elevated levels of distress (≥5) to express higher needs [10, 13, 28]. However, previous studies were all cross-sectional, did not adjust for multiple testing which may have overestimated their findings, and mainly included breast and prostate cancer patients, which limits the generalizability. Even though our sample shows high heterogeneity in cancer types, it is nevertheless not representative of the cancer population. The results must be interpreted with caution given this limitation.

We found the intention to use psychosocial services to be associated with the PHQ-9 and GAD-7, but not the DT. Only one previous study evaluated the association of patient intention and a screening instrument used in our study [20]. It showed the DT cut-off to be related to intention, however in a cross-sectional study design. One explanation for our contrasting results could be our longitudinal design and the differentiated assessment of various psychosocial services. There is to date no study evaluating the PHQ-9 and GAD-7 in relation to intention and our results are thus the first to show this association.

In line with our pilot study and a study with 84 head and neck cancer patients, we found that utilization of psychosocial support was not predicted by any of the screeners [12, 29]. In contrast, a cross-sectional study with 1398 cancer patients of heterogeneous diagnoses showed the DT, PHQ-9, and GAD-7 values to be associated with higher utilization of psychological care [19]. Several explanations are possible for these contrasting results. The previous study had a larger sample so that smaller effects could be detected, did not adjust for multiple testing, and was cross-sectional in design, which only permits conclusions about associations, but not about the predictive effects of the screeners.

### Clinical implications

The PHQ-9 and GAD-7 screeners investigated in this study were developed to assess psychological symptoms based on diagnostic criteria and were originally not intended to predict the willingness to seek psychosocial support for cancer patients. However, they are often used in clinical care planning to identify patients in objective need for support and to offer support accordingly. Our study does not aim to evaluate the quality of screeners, but to investigate their predictive value when used for clinical care planning. Our results imply that both screeners used as sum score or cut-off may facilitate supportive care planning. Especially the cut-offs of the screeners are quick and clear to interpret and can be useful for any medical staff in identifying patients in potential need or with potential intention to seek psychosocial support. The one-item tool DT for general distress is often used during the disease trajectory to consider further support. Our results imply that it can be used in clinical care planning to identify patients in need. However, the clinical information of one item is sparse and additional information need to be assessed to adequately plan support offers.

Nevertheless, not all patients with high levels of distress report a desire for help, and conversely, patients with low levels of distress may indeed express a need for further support [10, 11, 28, 30, 31]. Screening results thus represent an important contribution to psychosocial needs, but further aspects need to be assessed and considered in supportive care planning. Further sociodemographic variables that are associated with higher needs, e.g. female sex [28, 32, 33] and younger age [11, 28, 33, 34], as well as the assessment of the subjective need for psychosocial support might be important.

However, our results suggest that the screeners might not be adequate in predicting the actual utilization of psychosocial support services. This finding is plausible regarding the content of the screeners. Since the PHQ-9 and GAD-7 assess symptoms of depression and anxiety, patients with high values on the screeners may not seek support simply due to the burdensome character of their psychological symptomatology. Also, when taking into account organizational aspects of the reality of clinical care, barriers that may impede utilization of support may explain this result. Since we did not assess reasons for non-utilization, our results are limited in exploring the discrepancy between needs and utilization. Barriers can be either intrapersonal (e.g., patient reluctance [35], stigmatization, being too burdened to seek support, enough support from family and friends [19]) or organizational (e.g., distance to institutions, timing of support [36], lack of availability [37], or information about the services [19]). Consequently, informational material and normalization of psychosocial support during cancer may increase service utilization. In addition, the need and the intention to seek for support cannot be treated as a stable personality trait, but can change over time depending on the disease progression, symptom severity, or changes in roles within the family. Repeated offering for support across the disease trajectory thus appear suitable and necessary to increase the uptake of support services.

The screeners’ focus primarily on psychosocial distress aligns well with our findings, as the screeners were best in predicting psycho-oncological support service.

### Strengths

The main strength of our study is the longitudinal design that allows for a prediction of the willingness to seek psychosocial support during clinical cancer care. As we included different distress screeners, our data allows for a systematic comparison of these. Furthermore, our study is the first that includes all dimensions of willingness to seek psychosocial services in one study, thus capturing all stages from reporting a need, to the intention to use support services, and finally the actual utilization. We further applied a rigorous statistical approach by adjusting for multiple testing in order to reduce the risk of false positive results and sensitivity analyses to obtain robust conclusions.

### Limitations

Due to a high rate of non-responders, a selection bias is possible with regard to patients of better physical or mental condition and higher motivation to participate in the study. Unfortunately, no non-responder analysis was possible as medical data was not available for all patients. Even though the sample shows high heterogeneity with regard to cancer types, some cancer types are highly represented over others, which limits the generalizability of our results. The drop-out rate during the study was relatively high, which may have caused an attrition bias. Drop-outs showed higher distress at baseline and differed in medical data. This could have an impact on reported needs and utilization of psychosocial support. However, a sensitivity analysis according to the central medical variable in which drop-outs differed was run (palliative vs. curative), showing consistent results in both groups. Thus, the results can be interpreted as relatively robust. Furthermore, due to the study design with an intervention group receiving recommendations for psychosocial support, the effect of the screeners on the willingness to seek psychosocial support might have been overestimated. Again, sensitivity analysis among the control patients indicated a similar pattern of results, indicating a low risk of bias.

## Conclusion

This study demonstrated the predictive effect of several distress screeners on the need and intention to seek psychosocial support. Therefore, they could be useful as an important contributing factor in clinical cancer care planning. However, the utilization of support services was not predicted by established distress screeners. Research on barriers and supporting factors for the utilization of support services seems needed.

## Supplementary information

Supplementary file 1 Table S1. Sensitivity analysis of the control group only. Predictive effect of the three screeners as sum score values on service use by dimensions of willingness and types of services at t1 and t2, Table S2. Sensitivity analysis of curative cancer patients. Predictive effect of the three screeners as sum score values on service use by dimensions of willingness and types of services at t1 and t2, Table S3. Sensitivity analysis of palliative cancer patients. Predictive effect of the three screeners as sum score values on service use by dimensions of willingness and types of services at t1 and t2.

## Data Availability

The data and the code that support the findings are available upon request from the authors.
