# Supplementary material for: Effect of depression, anxiety, and distress screeners on the need, intention, and utilization of psychosocial support services among cancer patients
Source: Support Care Cancer. 2023 Jan 16;31(2):117. doi: 10.1007/s00520-023-07580-2 (PMC9842579; doi:10.1007/s00520-023-07580-2)
Supplement: Supplementary file 1 — Table S1. Sensitivity analysis of the control group only. Predictive effect of the three screeners as sum score values on service use by dimensions of willingness and types of services at t1 and t2, Table S2. Sensitivity analysis of curative cancer patients. Predictive effect of the three screeners as sum score values on service use by dimensions of willingness and types of services at t1 and t2, Table S3. Sensitivity analysis of palliative cancer patients. Predictive effect of the three screeners as sum score values on service use by dimensions of willingness and types of services at t1 and t2. [file 520_2023_7580_MOESM1_ESM.docx]

**Supplementary Information**

**Title**: Effect of depression, anxiety and distress screeners on the need, intention and utilization of psychosocial support services among cancer patients

**Journal**: Supportive Care in Cancer

**Authors**: Franziska Springer, Leon Sautier, Georgia Schilling, Uwe Koch-Gromus, Carsten Bokemeyer, Michael Friedrich, Anja Mehnert-Theuerkauf, Peter Esser

**Corresponding Author**: Franziska Springer, Department of Medical Psychology and Medical Sociology, University Medical Center Leipzig, Leipzig, Germany, E-Mail: franziska.springer@medizin.uni-leipzig.de

| Table S1. Sensitivity analysis of the control group only. Predictive effect of the three screeners as sum score values on service use by dimensions of willingness and types of services at t1 and t2 | | | | | | | | | | |  |
| --- | --- | --- | --- | --- | --- | --- | --- | --- | --- | --- | --- |
|  |  | **t1 (3 months follow-up)** | | | |  | **t2 (6 months follow-up)** | | | |  |
|  |  | ***N*** | ***Β (SE)*** | ***p^a^*** | ***OR*** |  | ***N*** | ***Β (SE)*** | ***p^a^*** | ***OR*** | |
| *Need* | |  | | | | | | | | |  |
| PHQ-9 | *Social Service* | 184 | 0.08 (0.04) | **.024** | 1.09 |  | 131 | 0.13 (0.05) | **.003** | 1.14 | |
|  | *Psycho-onco* | 184 | 0.12 (0.04) | **.001** | 1.13 |  | 136 | 0.13 (0.04) | **.002** | 1.14 | |
|  | *Survivorship* | 184 | 0.12 (0.04) | **.005** | 1.12 |  | 131 | 0.01 (0.06) | .918 | 1.01 | |
| GAD-7 | *Social Service* | 185 | 0.13 (0.04) | **.004** | 1.14 |  | 133 | 0.18 (0.06) | **.002** | 1.19 | |
|  | *Psycho-onco* | 185 | 0.18 (0.05) | **<.001** | 1.20 |  | 138 | 0.19 (0.06) | **<.001** | 1.21 | |
|  | *Survivorship* | 185 | 0.12 (0.05) | **.010** | 1.13 |  | 132 | 0.08 (0.06) | .179 | 1.09 | |
| DT | *Social Service* | 179 | 0.15 (0.08) | .060 | 1.16 |  | 125 | 0.11 (0.09) | .217 | 1.12 | |
|  | *Psycho-onco* | 179 | 0.21 (0.08) | **.010** | 1.23 |  | 130 | 0.16 (0.09) | .072 | 1.17 | |
|  | *Survivorship* | 179 | 0.23 (0.09) | **.013** | 1.26 |  | 125 | 0.09 (0.11) | .421 | 1.09 | |
| *Intention* | |  | | | | | | | | |  |
| PHQ-9 | *Social Service* | 166 | 0.09 (0.04) | **.014** | 1.10 |  | 125 | 0.10 (0.05) | **.030** | 1.10 | |
|  | *Psycho-onco* | 166 | 0.10 (0.04) | **.009** | 1.11 |  | 129 | 0.12 (0.04) | **.004** | 1.13 | |
|  | *Survivorship* | 164 | 0.09 (0.04) | **.021** | 1.10 |  | 129 | 0.08 (0.04) | .095 | 1.08 | |
| GAD-7 | *Social Service* | 167 | 0.15 (0.05) | **.001** | 1.16 |  | 127 | 0.11 (0.05) | **.035** | 1.12 | |
|  | *Psycho-onco* | 167 | 0.13 (0.05) | **.004** | 1.14 |  | 131 | 0.11 (0.04) | **.033** | 1.11 | |
|  | *Survivorship* | 165 | 0.10 (0.05) | **.033** | 1.10 |  | 130 | 0.06 (0.05) | .267 | 1.06 | |
| DT | *Social Service* | 161 | 0.11 (0.08) | .173 | 1.11 |  | 119 | -0.02 (0.10) | .798 | 0.98 | |
|  | *Psycho-onco* | 161 | 0.08 (0.08) | .320 | 1.08 |  | 123 | 0.11 (0.09) | .202 | 1.11 | |
|  | *Survivorship* | 159 | 0.13 (0.08) | .112 | 1.14 |  | 123 | 0.09 (0.10) | .345 | 1.09 | |
| *Utilization* | |  | | | | | | | | |  |
| PHQ-9 | *Social Service* | 190 | 0.02 (0.04) | .607 | 1.02 |  | 152 | -0.02 (0.04) | .628 | 0.98 | |
|  | *Psycho-onco* | 193 | 0.01 (0.04) | .079 | 1.01 |  | 152 | 0.07 (0.05) | .153 | 1.07 | |
|  | *Survivorship* | 188 | -0.28 (0.21) | .186 | 0.76 |  | 152 | 0.19 (0.16) | .230 | 1.21 | |
| GAD-7 | *Social Service* | 191 | 0.01 (0.04) | .735 | 1.01 |  | 154 | 0.04 (0.05) | .456 | 1.04 | |
|  | *Psycho-onco* | 194 | 0.00 (0.05) | .980 | 1.00 |  | 154 | 0.08 (0.05) | .139 | 1.08 | |
|  | *Survivorship* | 189 | -0.31 (0.29) | .281 | 0.73 |  | 154 | 0.44 (0.31) | .163 | 1.55 | |
| DT | *Social Service* | 185 | 0.13 (0.08) | .095 | 1.13 |  | 146 | 0.13 (0.09) | .140 | 1.14 | |
|  | *Psycho-onco* | 188 | 0.12 (0.08) | .120 | 1.13 |  | 146 | 0.21 (0.10) | **.041** | 1.23 | |
|  | *Survivorship* | 183 | -0.31 (0.32) | .342 | 0.74 |  | 146 | 0.73 (0.62) | .235 | 2.08 | |
| Note.  All regressions are controlled for age and gender  B, unstandardized regression coefficient; SE, standard error; p, unadjusted p-value of the regression model; OR, odds-ratio  ^a^ no Bonferroni-Holm adjustment was made due to reduced sample size | | | | | | | | | | |  |

| Table S2. Sensitivity analysis of curative cancer patients. Predictive effect of the three screeners as sum score values on service use by dimensions of willingness and types of services at t1 and t2 | | | | | | | | | | |  |
| --- | --- | --- | --- | --- | --- | --- | --- | --- | --- | --- | --- |
|  |  | **t1 (3 months follow-up)** | | | |  | **t2 (6 months follow-up)** | | | |  |
|  |  | ***N*** | ***Β (SE)*** | ***p^b^*** | ***OR*** |  | ***N*** | ***Β (SE)*** | ***p^b^*** | ***OR*** | |
| *Need* | |  | | | | | | | | |  |
| PHQ-9^a^ | *Social Service* | 146 | 0.09 (0.03) | **.010** | 1.09 |  | 115 | 0.12 (0.04) | **.005** | 1.13 | |
|  | *Psycho-onco* | 146 | 0.08 (0.04) | **.037** | 1.08 |  | 118 | 0.14 (0.04) | **.001** | 1.15 | |
|  | *Survivorship* | 146 | 0.06 (0.04) | .081 | 1.07 |  | 112 | 0.09 (0.05) | .069 | 1.10 | |
| GAD-7^a^ | *Social Service* | 146 | 0.10 (0.04) | **.019** | 1.10 |  | 115 | 0.15 (0.05) | **.005** | 1.16 | |
|  | *Psycho-onco* | 146 | 0.10 (0.04) | **.018** | 1.11 |  | 118 | 0.19 (0.05) | **<.001** | 1.21 | |
|  | *Survivorship* | 146 | 0.04 (0.04) | .419 | 1.04 |  | 112 | 0.22 (0.07) | **.001** | 1.24 | |
| DT^a^ | *Social Service* | 143 | 0.16 (0.08) | **.042** | 1.17 |  | 111 | 0.12 (0.10) | .197 | 1.13 | |
|  | *Psycho-onco* | 143 | 0.27 (0.09) | **.003** | 1.31 |  | 114 | 0.28 (0.10) | **.007** | 1.33 | |
|  | *Survivorship* | 143 | -0.01 (0.08) | .934 | 0.99 |  | 108 | 0.23 (0.12) | .060 | 1.25 | |
| *Intention* | |  | | | | | | | | |  |
| PHQ-9^a^ | *Social Service* | 140 | 0.08 (0.04) | **.032** | 1.08 |  | 107 | 0.08 (0.04) | .070 | 1.08 | |
|  | *Psycho-onco* | 140 | 0.11 (0.04) | **.006** | 1.11 |  | 110 | 0.13 (0.05) | **.004** | 1.14 | |
|  | *Survivorship* | 140 | 0.07 (0.04) | .063 | 1.07 |  | 108 | 0.10 (0.04) | **.026** | 1.10 | |
| GAD-7^a^ | *Social Service* | 140 | 0.07 (0.04) | .099 | 1.07 |  | 107 | 0.09 (0.05) | .085 | 1.09 | |
|  | *Psycho-onco* | 140 | 0.09 (0.05) | **.048** | 1.09 |  | 110 | 0.16 (0.05) | **.003** | 1.17 | |
|  | *Survivorship* | 140 | 0.04 (0.04) | .324 | 1.04 |  | 108 | 0.11 (0.05) | **.034** | 1.11 | |
| DT^a^ | *Social Service* | 137 | 0.06 (0.08) | .404 | 1.07 |  | 103 | 0.02 (0.10) | .837 | 1.02 | |
|  | *Psycho-onco* | 137 | 0.07 (0.08) | .374 | 1.08 |  | 106 | 0.21 (0.10) | **.037** | 1.23 | |
|  | *Survivorship* | 137 | 0.04 (0.08) | .599 | 1.04 |  | 104 | 0.18 (0.10) | .080 | 1.20 | |
| *Utilization* | |  | | | | | | | | |  |
| PHQ-9^a^ | *Social Service* | 150 | 0.05 (0.04) | .156 | 1.05 |  | 122 | 0.00 (0.04) | .946 | 1.00 | |
|  | *Psycho-onco* | 151 | 0.08 (0.04) | .053 | 1.08 |  | 121 | 0.05 (0.05) | .315 | 1.05 | |
|  | *Survivorship* | 145 | -0.14 (0.11) | .182 | 0.87 |  | 121 | 0.03 (0.13) | .829 | 1.03 | |
| GAD-7^a^ | *Social Service* | 150 | 0.01 (0.04) | .839 | 1.01 |  | 122 | 0.07 (0.05) | .148 | 1.07 | |
|  | *Psycho-onco* | 151 | 0.08 (0.05) | .068 | 1.09 |  | 121 | 0.04 (0.05) | .439 | 1.04 | |
|  | *Survivorship* | 145 | -0.12 (0.13) | .341 | 0.89 |  | 121 | 0.11 (0.13) | .407 | 1.11 | |
| DT^a^ | *Social Service* | 147 | 0.15 (0.09) | .058 | 1.16 |  | 118 | 0.12 (0.09) | .163 | 1.13 | |
|  | *Psycho-onco* | 148 | 0.16 (0.09) | .081 | 1.17 |  | 117 | 0.13 (0.11) | .246 | 1.13 | |
|  | *Survivorship* | 142 | -0.09 (0.18) | .602 | 0.91 |  | 117 | 0.02 (0.30) | .937 | 1.02 | |
| Note.  All regressions are controlled for the intervention, age and gender  B, unstandardized regression coefficient; SE, standard error; p, unadjusted p-value of the regression model; OR, odds-ratio  ^a^ no Bonferroni-Holm adjustment was made due to reduced sample size | | | | | | | | | | |  |

| Table S3. Sensitivity analysis of palliative cancer patients. Predictive effect of the three screeners as sum score values on service use by dimensions of willingness and types of services at t1 and t2 | | | | | | | | | | |  |
| --- | --- | --- | --- | --- | --- | --- | --- | --- | --- | --- | --- |
|  |  | **t1 (3 months follow-up)** | | | |  | **t2 (6 months follow-up)** | | | |  |
|  |  | ***N*** | ***Β (SE)*** | ***p^b^*** | ***OR*** |  | ***N*** | ***Β (SE)*** | ***p^b^*** | ***OR*** | |
| *Need* | |  | | | | | | | | |  |
| PHQ-9^a^ | *Social Service* | 141 | 0.12 (0.04) | **.005** | 1.13 |  | 81 | 0.19 (0.06) | **.003** | 1.21 | |
|  | *Psycho-onco* | 141 | 0.13 (0.04) | **.002** | 1.14 |  | 81 | 0.14 (0.06) | **.012** | 1.16 | |
|  | *Survivorship* | 141 | 0.11 (0.05) | **.020** | 1.12 |  | 80 | -0.05 (0.08) | .498 | 0.95 | |
| GAD-7^a^ | *Social Service* | 141 | 0.17 (0.05) | **.001** | 1.18 |  | 82 | 0.25 (0.08) | **.002** | 1.28 | |
|  | *Psycho-onco* | 141 | 0.21 (0.05) | **<.001** | 1.23 |  | 82 | 0.19 (0.07) | **.007** | 1.21 | |
|  | *Survivorship* | 141 | 0.18 (0.06) | **.003** | 1.20 |  | 81 | -0.02 (0.09) | .803 | 0.98 | |
| DT^a^ | *Social Service* | 139 | 0.18 (0.10) | .062 | 1.20 |  | 79 | 0.34 (0.14) | .014 | 1.41 | |
|  | *Psycho-onco* | 139 | 0.18 (0.09) | .054 | 1.19 |  | 79 | 0.19 (0.12) | .127 | 1.20 | |
|  | *Survivorship* | 139 | 0.49 (0.14) | **.001** | 1.63 |  | 78 | -0.33 (0.17) | .050 | 0.72 | |
| *Intention* | |  | | | | | | | | |  |
| PHQ-9^a^ | *Social Service* | 128 | 0.09 (0.04) | **.035** | 1.09 |  | 74 | 0.14 (0.06) | **.027** | 1.15 | |
|  | *Psycho-onco* | 128 | 0.11 (0.04) | **.015** | 1.11 |  | 74 | 0.18 (0.07) | **.009** | 1.19 | |
|  | *Survivorship* | 126 | 0.09 (0.05) | **.048** | 1.10 |  | 74 | 0.07 (0.06) | .244 | 1.07 | |
| GAD-7^a^ | *Social Service* | 128 | 0.15 (0.05) | **.003** | 1.17 |  | 75 | 0.18 (0.07) | **.015** | 1.19 | |
|  | *Psycho-onco* | 128 | 0.14 (0.05) | **.008** | 1.15 |  | 75 | 0.13 (0.07) | .070 | 1.14 | |
|  | *Survivorship* | 126 | 0.11 (0.05) | **.036** | 1.11 |  | 75 | 0.09 (0.07) | .231 | 1.09 | |
| DT^a^ | *Social Service* | 126 | 0.10 (0.09) | .285 | 1.11 |  | 72 | 0.17 (0.14) | .218 | 1.18 | |
|  | *Psycho-onco* | 126 | 0.12 (0.10) | .226 | 1.12 |  | 72 | 0.33 (0.15) | **.029** | 1.39 | |
|  | *Survivorship* | 124 | 0.26 (0.12) | **.027** | 1.29 |  | 72 | -0.06 (0.13) | .654 | 0.94 | |
| *Utilization* | |  | | | | | | | | |  |
| PHQ-9^a^ | *Social Service* | 146 | 0.02 (0.04) | .595 | 1.02 |  | 96 | 0.03 (0.05) | .612 | 1.03 | |
|  | *Psycho-onco* | 146 | 0.07 (0.04) | .061 | 1.08 |  | 96 | 0.12 (0.05) | **.027** | 1.13 | |
|  | *Survivorship* | 143 | -0.09 (0.29) | .778 | 0.92 |  | 95 | -0.12 (0.35) | .738 | 0.89 | |
| GAD-7^a^ | *Social Service* | 146 | 0.02 (0.05) | .725 | 1.02 |  | 97 | 0.03 (0.06) | .608 | 1.03 | |
|  | *Psycho-onco* | 146 | 0.08 (0.05) | .077 | 1.09 |  | 97 | 0.21 (0.07) | **.003** | 1.24 | |
|  | *Survivorship* | 143 | -0.22 (0.53) | .675 | 0.80 |  | 96 | -0.32 (0.60) | .602 | 0.73 | |
| DT^a^ | *Social Service* | 144 | 0.11 (0.10) | .240 | 1.12 |  | 94 | 0.19 (0.11) | .098 | 1.21 | |
|  | *Psycho-onco* | 144 | 0.23 (0.09) | **.017** | 1.25 |  | 94 | 0.49 (0.15) | **.001** | 1.63 | |
|  | *Survivorship* | 141 | -0.56 (0.70) | .423 | 0.57 |  | 93 | -0.62 (0.76) | .420 | 0.54 | |
| Note.  All regressions are controlled for the intervention, age and gender  B, unstandardized regression coefficient; SE, standard error; p, unadjusted p-value of the regression model; OR, odds-ratio  ^a^ no Bonferroni-Holm adjustment was made due to reduced sample size | | | | | | | | | | |  |
